# Gadolinium-based contrast agent-free (GBCA-free) versus gadolinium-based contrast agent-enhanced (GBCA-enhanced) magnetic resonance imaging as a screening tool for intracranial tumors

**DOI:** 10.3389/fnimg.2026.1776934

**Published:** 2026-04-28

**Authors:** Haider Faeq Hadhratee Al-Rubaiee, Samr Adnan Abdaljabbar Al-Salm, Weronika Jensen, Agnieszka Monika Delekta, Yousef Yavarian, Boris Modrau

**Affiliations:** 1 Aalborg University Hospital, Aalborg, Denmark; 2 Aarhus University Hospital, Aarhus, Denmark

**Keywords:** brain tumor, GBCA, MRI, non-GBCA, neuroradiology

## Abstract

**Background:**

GBCA-enhanced MRI is considered the gold standard for diagnosing intracranial tumors. However, concerns regarding gadolinium-based contrast agents (GBCAs) and workflow limitations have led to increasing interest in GBCA-free MRI techniques.

**Objective:**

To compare the diagnostic performance of GBCA-free MRI with GBCA-enhanced MRI for detecting intracranial tumors within a neurological diagnostic pathway.

**Methods:**

In this retrospective cohort study, 191 patients with 195 tumors (124 intra-axial and 71 extra-axial) and 410 controls were included from a regional brain tumor diagnostic pathway between 2013 and 2022. Three senior neuroradiologists independently assessed anonymized MRI scans. Each scan was first evaluated using GBCA-free sequences and subsequently reassessed using the full GBCA-enhanced protocol. Sensitivity, specificity, and inter-method agreement were calculated.

**Results:**

GBCA-free MRI demonstrated a sensitivity of 79.6% and a specificity of 96.1%, whereas GBCA-enhanced MRI showed a sensitivity of 73.3% and a specificity of 92.9% (*p* = 0.15). Agreement between the two imaging approaches was substantial (*κ* = 0.699). Subgroup analyses showed comparable performance for intra-axial tumors, while both imaging approaches demonstrated modest sensitivity for extra-axial tumors.

**Conclusion:**

GBCA-free MRI demonstrated diagnostic performance comparable to that of GBCA-enhanced MRI for detecting intracranial tumors. These findings suggest that GBCA-free MRI may serve as a reliable first-line screening tool within the neurological diagnostic pathway, reserving GBCA administration for cases requiring further lesion characterization.

## Introduction

Screening and further assessment of intracranial tumors is critically dependent on advanced imaging modalities, with magnetic resonance imaging (MRI) being the gold standard for neuroimaging. Traditionally, contrast-enhanced MRI, or better described gadolinium-based contrast agents (GBCAs), has been the preferred approach due to its superior ability to delineate tumor margins and enhance lesion visibility in both intra-axial and extra-axial tumors (Jung et al., 2012). However, concerns regarding the potential side effects of contrast agents, such as nephrogenic systemic fibrosis (NSF) in patients with renal impairment (van der Molen, 2014) and the deposition of gadolinium in brain tissue following repeated exposure (Rogosnitzky and Branch, 2016).

In response to these concerns, non-contrast-enhanced imaging techniques, more correctly GBCA-free, have gained increasing attention, especially in settings where MRI is performed on many patients without assessing renal function due to accelerated workflow. Recent studies suggest that these GBCA-free techniques may be non-inferior in certain clinical scenarios, providing high diagnostic accuracy while eliminating the risks associated with contrast media (Eichinger et al., 2019).

The Danish National Board of Health has developed specific criteria for screening patients for primary brain tumors to facilitate timely diagnosis and treatment. Patients are considered for the diagnostic pathway if they exhibit one of several key symptoms, including recent focal neurological deficits without other causes, first-time seizures in adults, unexplained behavioral or cognitive changes, new, progressively worsening headaches. These criteria are designed to ensure that patients with potential intracranial tumors are promptly referred for further imaging diagnostics (Al-Rubaiee and Modrau, 2025). The current diagnostic pathway relies heavily on GBCA-enhanced MRI in region of North Denmark. This approach may increase the use of gadolinium-based contrast agents even in patients ultimately found not to have intracranial pathology. Furthermore, in settings with limited imaging resources or high referral volumes, the requirement for contrast-enhanced MRI may contribute to workflow delays and increased healthcare costs.

### Aim of the study

This study aims to evaluate the sensitivity and specificity of GBCA-free MRI sequences in screening intracranial tumors compared to the complete standard GBCA-enhanced MRI protocol.

## Methods

In alignment with referring criteria, the recommended work-up in North Denmark Region includes a telephone consultation between the referring physician and the neurologist on call at Aalborg University Hospital. If the neurologist concurs that the presenting symptoms could be due to an intracranial tumor, a direct referral for an MRI scan of the brain with GBCA is ordered, provided there are no contraindications (e.g., pacemakers). Otherwise, a CT scan of the brain is performed. The imaging must be completed within 6 days from the date of referral in accordance with national guidelines (Sundhed.dk, 2023).

The assessed MR images in our study are part of a quality control study of the brain tumor pathway performed by the neurology department at Aalborg University Hospital (Al-Rubaiee and Modrau, 2025), where 2,055 patients were originally referred with suspicion of a brain tumor between 2013 and 2022. Of these, neuroradiologists identified 207 patients with an intracranial tumor. The assessment was based on clinical information from the referral, MRI with GBCA, and all available clinical data and previous imaging. These original reports were defined as the gold standard, as they were evaluated with access to full clinical data, previous radiological imaging material, and usually peer review by a multi-disciplinary team.

In this retrospective cohort study, we included all tumor cases after excluding 16 patients with hypophyseal tumors, resulting in 191 patients. Among these, four patients had both intra-axial and extra-axial tumors simultaneously, leading to a total of 195 tumors (124 intra-axial and 71 extra-axial). A control group of 410 patients (maintaining an approximately 1:2 tumor-to-control ratio) was randomly selected from the remaining 1,848 patients without reported intracranial tumors. The scans of these 601 patients were anonymized, and randomly divided into three groups (with 204, 197, and 200 scans, respectively). Each group was assessed by a senior neuroradiologist, who was blinded to all clinical and demographic data. All randomization was performed using Stata 18 software.

For each case (tumor or control), the neuroradiologists were initially exposed only to the GBCA-free imaging set, which included axial diffusion-weighted imaging (DWI) with *b*-values of 0 and 1,000 (TE/TR = 70/8,000 ms), sagittal T2 (TE/TR = 120/5,500 ms), and coronal T2-FLAIR (TE/TR = 120/8,500 ms). The presence or absence of an intracranial tumor and its location (intra-axial or extra-axial) were recorded. In the second round, the neuroradiologists were provided with the entire scan set, but in a randomly different order than in GBCA-free set. The entire set included the GBCA-free sequences along with GBCA-enhanced MRI sequences. A dose of 0.2 mL/kg gadoteridol (ProHance^®^, Bracco Imaging, Milan, Italy) was administered, with axial T1-FLAIR (TE/TR = 10/600 ms) acquired 7 min post-contrast and coronal T1-FLAIR (TE/TR = 10/600 ms) acquired 10 min post-contrast. The slice thickness was 5 mm for all sequences.

Also in this second round, the presence or absence of an intracranial tumor and its location were documented, while the neuroradiologists were blinded for the first-round assessments. The size of tumors was not investigated in our study, because our study focuses mainly on comparing the efficacy of the two above methods.

Intracranial tumor was defined as any focal intracranial lesion demonstrating imaging characteristics suggestive of neoplasia, including mass effect, abnormal signal intensity on T2-weighted or FLAIR sequences, restricted diffusion on DWI when present, or GBCA-enhancement patterns consistent with neoplastic lesions. Lesions suspected to represent vascular, inflammatory, or non-neoplastic abnormalities were not classified as tumors. If radiological diagnosis could not be made, further referral to new scanning was requested and those patients had no tumor diagnosis in the initial study and were not part of the 195-tumor group.

### Statistical analysis

Statistical analysis was performed using STATA 18 (StataCorp 2023, Stata Statistical Software: Release 18. College Station, TX: StataCorp LLC). Sensitivity and specificity for detecting intracranial tumors as whole, as well as for intra-axial and extra-axial tumors, were calculated for each neuroradiologist as well as for the combined evaluation, confidence intervals were calculated using the Wilson score method. For the primary endpoint detection of intracranial tumor, the McNemar’s test was used to compare the performance in terms of sensitivity of GBCA-enhanced versus GBCA-free imaging. Inter-method agreement was assessed using Cohen’s kappa coefficient, quantifying the level of agreement between the two imaging techniques beyond chance. Inter-rater reliability analysis between the 3 raters was not performed due to the fact that each group was assessed by only one neuroradiologist and therefore not possible to perform neither McNemar’s test nor Cohen’s kappa coefficient test between the 3 assessed groups.

We did not apply McNemar’s test or Cohen’s kappa for the secondary analyses of intra-axial and extra-axial tumors. These subgroups were evaluated independently, and the aim was to descriptively compare diagnostic performance rather than test for statistically significant differences or inter-rater agreement. McNemar’s test requires paired binary outcomes, which was not the case in our subgroup analysis, as the presence of intra- and extra-axial tumors varies across patients and was not evaluated as a dichotomous decision. Similarly, Cohen’s kappa was not used due to its sensitivity to prevalence and its limited interpretability in smaller or imbalanced subgroups. Instead, we focused on reporting sensitivity and specificity for each modality to reflect their respective diagnostic performance in real-world clinical conditions.

A formal sample size calculation was not performed because the study was based on an existing cohort from a quality control study of the regional brain tumor diagnostic pathway. All eligible tumor cases within the study period were included.

## Results

In the combined evaluation of all 3 raters, non-contrast MRI demonstrated a sensitivity of 79.6% (95% CI: 73.3–84.7%) and specificity of 96.1% (95% CI: 93.8–97.6%) compared to contrast-enhanced MRI with a sensitivity of 73.3% (95% CI: 66.6–79.1%) and specificity of 92.9% (95% CI: 90.0–95.0%). This was not statistically significant (*p* = 0.15), odds ratio of 1.706, 95% confidence interval includes 1 (0.907 to 3.309).

The agreement between GBCA-enhanced and GBCA-free imaging reflecting the reliability in detecting brain tumors, assessed by the Cohen’s kappa analysis, was *κ* = 0.699. Table 1 provides further details.

**Table 1:** Intracranial tumors assessed by GBCA-free MRI versus GBCA-enhanced MRI.

Raters	Intracranial tumors detected by non-contrast MRI						Intracranial tumors detected by contrast MRI					
	TP	FP	TN	FN	Sensitivity	Specificity	TP	FP	TN	FN	Sensitivity	Specificity
Rater 1 (*n* = 204)	55	7	131	11	83.3%	94.9%	53	5	133	13	80.3%	96.4%
Rater 2 (*n* = 197)	47	0	136	14	77%	100%	50	2	134	11	81.9%	98.5%
Rater 3 (*n* = 200)	50	9	127	14	78.1%	93.4%	37	22	114	27	57.8%	83.8%
Rater 1–3 (*n* = 601)	152	16	394	39	79.6%	96.1%	140	29	381	51	73.3%	92.9%

Number of intracranial tumors detected by the neuroradiologists when only GBCA-free images are evaluated, and then when GBCA-enhanced images are added to the evaluation, compared to the original reports. (*n*, number of patients; TP, true positive; FP, false positive; TN, true negative; FN, false negative). Sensitivity and specificity were calculated accordingly.

As a secondary endpoint, we analyzed the performance of GBCA-free imaging in detecting intra-axial and extra-axial tumors. For intra-axial tumors, GBCA-free MRI showed a sensitivity of 79.0% (95% CI: 71.0–85.3%) and specificity of 98.5% (95% CI: 97.0–99.3%), while GBCA-enhanced MRI demonstrated a sensitivity of 74.2% (95% CI: 65.8–81.1%) and specificity of 96.2% (95% CI: 94.1–97.6%). For extra-axial tumors, GBCA-free MRI had a sensitivity of 56.3% (95% CI: 44.8–67.3%) and specificity of 95.3% (95% CI: 93.1–96.8%), whereas GBCA-enhanced MRI yielded a sensitivity of 57.8% (95% CI: 46.2–68.5%) and specificity of 96.2% (95% CI: 94.2–97.5%). Tables 2, 3 provide further details.

**Table 2:** Intra-versus extra-axial tumors assessed by GBCA-free MRI.

Raters	Intra-axial tumors detected by non-contrast MRI						Extra-axial tumors detected by non-contrast MRI					
	TP	FP	TN	FN	Sensitivity	Specificity	TP	FP	TN	FN	Sensitivity	Specificity
Rater 1 (*n* = 204)	35	3	159	7	83.3%	98.1%	15	11	169	9	62.5%	93.9%
Rater 2 (*n* = 197)	27	0	160	10	72.9%	100%	14	6	166	11	58.3%	96.53%
Rater 3 (*n* = 200)	36	4	251	9	80%	98.43%	11	8	170	11	50.0%	95.51%
Rater 1–3 (*n* = 601)	98	7	470	26	79.03%	98.53%	40	25	505	31	56.34%	95.28%

Number of tumors detected by the neuroradiologists when only GBCA-free MRI was used, compared to the original reports. (*n*, number of patients; TP, true positive; FP, false positive; TN, true negative; FN, false negative). Sensitivity and specificity were calculated accordingly.

**Table 3:** Intra-versus extra-axial tumors assessed by GBCA-enhanced MRI.

Raters	Intra-axial tumors detected by contrast MRI						Extra-axial tumors detected by contrast MRI					
	TP	FP	TN	FN	Sensitivity	Specificity	TP	FP	TN	FN	Sensitivity	Specificity
Rater 1 (*n* = 204)	38	2	160	4	90.48%	98.77%	14	5	175	10	58.33%	97.22%
Rater 2 (*n* = 197)	29	2	158	8	78.38%	98.75%	19	3	170	5	79.17%	98.27%
Rater 3 (*n* = 200)	25	14	141	20	55.56%	91.03%	8	12	165	15	34.78%	93.22%
Rater 1–3 (*n* = 601)	92	18	459	32	74.19%	96.49%	41	20	510	30	57.75%	96.23%

Number of tumors detected by the neuroradiologists when the whole set of the MRI was analyzed, compared to the original reports. (*n*, number of patients; TP, true positive; FP, false positive; TN, true negative; FN, false negative). Sensitivity and specificity were calculated accordingly.

## Discussion

This study compared the diagnostic performance of GBCA-free versus GBCA-enhanced MRI in detecting of intracranial tumors. The primary finding was that sensitivity of GBCA-free MRI imaging in detecting intracranial tumors was 79.6%, while its specificity was 96.1%. GBCA-enhanced MRI demonstrated a sensitivity of 73.3% and a specificity of 92.9% in detecting intracranial tumors without statistically significant difference. Furthermore, Cohen’s kappa analysis showed substantial agreement between the two methods in detecting tumors (*κ* = 0.699).

Our findings align with a similar study by Park et al. (2010), which demonstrated that GBCA-free imaging (using SWI and ADC) accurately diagnosed tumor pathology in 52 (71%) of 73 tumors. The lower sensitivity for detecting intracranial tumors, as well as the lack of 100% specificity compared to the clinical original MRI-reports, reflects the general challenge of evaluating intracranial tumors without clinical context. Our study evaluated only the radiological aspect of decision-making, without clinical context. We argue that signs and symptoms significantly influence the radiological interpretation, and additional peer review of a multidisciplinary team can alter the final neuroradiological diagnoses.

A possible explanation for the slightly higher sensitivity seen with GBCA-free MRI could be a kind of reverse satisfaction of search error, where the radiologist starts by evaluating GBCA-enhanced sequences and if no pathological enhancement is found, then the radiologist’s detection efficiency for the non-contrast sequences decreases. The true phenomenon is well-known as satisfaction of search error (Murphy et al., 2014), which is frequent and occurs in approximately 22% of diagnostic radiology.

Additionally, diffusion-weighted imaging can reveal cellular tumor components or associated ischemic changes. These signal abnormalities may sometimes be more conspicuous than subtle GBCA-enhancement, particularly when readers evaluate images without clinical context and that is why, it could explain relatively higher sensitivity observed with GBCA-free imaging.

As a secondary endpoint, we evaluated the diagnostic performance of GBCA-free versus GBCA-enhanced MRI in detecting intra-axial and extra-axial tumors. For intra-axial lesions, GBCA-free MRI demonstrated a sensitivity of 79.0% and specificity of 98.5%, compared to a sensitivity of 74.2% and specificity of 96.2% with GBCA-enhanced imaging. For extra-axial tumors, both modalities demonstrated lower sensitivity. GBCA-free MRI achieved a sensitivity of 56.3% and specificity of 95.3%, while GBCA-enhanced MRI showed a sensitivity of 57.8% and specificity of 96.2%.

These modest sensitivities in extra-axial tumors, despite high specificity, highlight the inherent challenges in interpreting the usual radiological work up. Lack of clinical context as well as no possibility to check previous imaging might play a role in the decision making in those assessments, and thus, the radiologists were less likely to describe any suspicious hyperintensity as tumor. We argue that this substantially contributed to the low sensitivity rates.

Previous data are limited in the literature and difficult to compare with our results. Murphy et al. (2014) reviewed the existing literature of using gadolinium-based contrast agents (GBCAs) in the diagnosis and treatment pathway of intracranial tumors and concluded that half-dose GBCAs for the follow up of gliomas and T2-weighted imaging alone for meningiomas are among options to reduce GBCA use without compromising the diagnostic accuracy of the imaging studies. This might support our findings that GBCA-free MRI can be an alternative to GBCA-enhanced MRI in certain diagnostic questions.

From a health-economic perspective, the use of GBCA-free MRI as an initial screening tool may offer potential advantages. Eliminating routine GBCA administration reduces the cost associated with gadolinium-based contrast agents and decreases examination time. In large diagnostic pathways such as the regional brain tumor pathway evaluated in this study, where the majority of referred patients do not ultimately have tumors, avoiding GBCA administration in the initial scan could result in substantial resource savings while maintaining diagnostic accuracy. Patients requiring further lesion characterization could subsequently undergo targeted GBCA-enhanced imaging.

To our knowledge, prospective studies have not been conducted, comparing the efficacy of GBCA-free based imaging in detecting brain tumors versus GBCA-enhanced images. A nonsystematic literature review conducted by Wamelink et al. (2024), included 51 articles, reviewing the use of GBCA-free imaging in the initial diagnostic of brain tumors concluded at reduced doses of GBCA could be considered in the initial diagnostic as well as skipping the use of GBCA in the post operative follow up of meningiomas. This is also line with our conclusion, that avoidance of GBCA can be safely used in the initial diagnostic of brain tumors.

The findings of our study indicate that GBCA-enhanced MRI did not outperform GBCA-free MRI in detecting intracranial tumors and this was also the case in the sub analysis of intra-axial and extra-axial intracranial tumors. This is of interest as GBCA-enhanced MRI is traditionally considered the gold standard for diagnostic work-up of intracranial tumors. Thus, GBCA-free MRI may serve as a reliable initial diagnostic tool, particularly in settings where GBCA administration is contraindicated, or as in our study, as a first step in a diagnostic pathway for intracranial tumors. This is of no harm, avoids potential side effects of GBCA, and allows a cost-effective and accelerated diagnostic pathway.

The strengths of our study include a large sample size of real-world data, which encompassed all patients diagnosed with intracranial tumors within the study period, as well as fully randomized and blinded assessment of the images. The primary limitation is that each scan was reviewed by only one neuroradiologist without access to clinical data and previous images. In real-life practice, MRI scans are typically interpreted in the context of a patient’s clinical presentation and additional peer review in a multidisciplinary team, which might influence diagnostic accuracy.

Future research should focus on prospective validation of GBCA-free MRI-based screening pathways. Prospective cohort studies could evaluate whether an initial GBCA-free MRI strategy influences clinically relevant outcomes such as diagnostic time, treatment delay, and patient prognosis. In addition, emerging artificial intelligence (AI)-based image analysis methods may enhance the detection of subtle lesions on GBCA-free MRI sequences. AI-assisted analysis of T2-FLAIR and diffusion-weighted images could potentially improve sensitivity for small or extra-axial tumors and further optimize GBCA-free screening protocols.

### Limitations

Several limitations of this study should be acknowledged. First, each MRI scan was evaluated by only one neuroradiologist, and the same cases were not independently assessed by multiple raters. Consequently, formal inter-rater reliability analysis between the three neuroradiologists could not be performed. Although this approach allowed for blinded evaluation of a large dataset, variability in individual interpretation may have influenced the observed diagnostic performance.

Second, the neuroradiologists assessed the anonymized MRI scans without access to clinical information, previous imaging studies, or multidisciplinary discussion. In routine clinical practice, MRI interpretation is typically performed in the context of the patient’s clinical presentation and may involve additional peer review, which could influence diagnostic accuracy.

Third, the study did not include detailed analysis of tumor size, lesion multiplicity, or tumor subtype. These factors may influence detectability on MRI, particularly for small metastases or leptomeningeal disease, which often rely on GBCA enhancement for optimal visualization. Future studies incorporating volumetric measurements and tumor classification would provide further insight into the diagnostic performance of GBCA-free MRI across different tumor types.

Fourth, the MRI protocol used in this study reflects the real-world clinical imaging pathway during the study period (2013–2022) and included two-dimensional sequences with a slice thickness of 5 mm. Contemporary neuro-oncological imaging often utilizes high-resolution three-dimensional post-GBCA-enhanced sequences with isotropic voxels, which may improve the detection of small lesions.

Fifth, the time required for image interpretation was not recorded. It is possible that evaluation of GBCA-free MRI alone may require longer reading time in some cases, which could influence workflow efficiency in clinical practice, but with clinical information, this limitation could be overcame.

Sixth, a *post-hoc* precision-based sample size assessment showed that approximately 250–301 tumor cases would have been required to estimate the observed sensitivities with a 95% confidence interval half-width of ±5%, whereas our study included 191 tumor cases. In contrast, only approximately 58–102 control cases would have been required to estimate the observed specificities with the same precision, and our study included 410 controls. Thus, the study was sufficiently sized for specificity estimates but less precise for sensitivity estimates, particularly in subgroup analyses.

Finally, the retrospective design of the study may introduce inherent biases related to patient selection and data availability. Prospective studies using standardized imaging protocols and including clinical outcome measures are needed to further validate the role of GBCA-free MRI as an initial screening tool in the diagnostic pathway for intracranial tumors.

## Conclusion

Our retrospective cohort study demonstrates that GBCA-free and GBCA-enhanced MRI have similar sensitivity and specificity in detection of intracranial tumors. Thus, we argue that GBCA-free MRI can serve as screening tool and GBCA-enhanced MRI can be reserved for cases requiring for small or subtle lesions and further lesion characterization. We encourage future research to explore tailored imaging protocols based on tumor subtypes and clinical presentation to optimize diagnostic efficiency while minimizing potentially harmful GBCA exposure.

## Data Availability

The datasets generated and/or analyzed during the current study are not publicly available due to institutional data protection policies but are available from the corresponding author on reasonable request. Requests to access these datasets should be directed to h.alrubaiee@rn.dk.
